# Fecundity and longevity of roaming dogs in Jaipur, India

**DOI:** 10.1186/1746-6148-4-6

**Published:** 2008-01-31

**Authors:** John F Reece, Sunil K Chawla, Elly F Hiby, Lex R Hiby

**Affiliations:** 1 Help in Suffering, Maharani Farm, Durgapura, Jaipur 302018 Rajasthan, India; 2 World Society for Protection of Animals, 89 Albert Embankment, London, SE1 7TP, UK; 3 Conservation Research Ltd., 110 Hinton Way, Gt. Shelford, Cambridge CB22 5AL, UK

## Abstract

**Background:**

Estimates of demographic parameters, such as age-specific survival and fecundity, age at first pregnancy and litter size, are required for roaming dogs (i.e. dogs that are neither confined nor restricted) to assess the likely effect of proposed methods of population control. Data resulting from individual identification of dogs spayed as part of an Animal Birth Control (ABC) programme in Jaipur, India, are used to derive such parameters for the roaming dog population of that city.

**Results:**

The percentage of females becoming pregnant in any given year was estimated by inspection of over 25,000 females caught for spaying from 1995 to 2006. The point estimate is 47.5% with a 95% confidence interval from 44% to 51%. Adult annual survival of spayed females was estimated by recapture of 62 spayed females from 2002 to 2006. The point estimate is 0.70 (95% confidence interval from 0.62 to 0.78), corresponding to an expected total lifespan of 3.8 years for a spayed female at one year old.

**Conclusion:**

Recording the pregnancy status of dogs collected for spaying and individual marking of dogs released following spaying can provide estimates of some of the demographic parameters essential for predicting the future effectiveness of an ABC programme. Further, we suggest that recording the number and location of spayed and unspayed dogs encountered by the catching teams could be the most effective way to monitor the size and composition of the roaming dog population.

## Background

For owned dogs, veterinary records can provide extensive data sets for the estimation of demographic parameters such as age-specific survival and fecundity, age at first pregnancy and litter size [e.g. [1]]. Similar estimates are required for roaming dogs (i.e. dogs that are neither confined nor restricted, sometimes known as "street", "stray" or "free-ranging" dogs), in particular to assess the likely effect of proposed methods of population control, but far less data are currently available for such dogs.

With the spread of urbanisation throughout the developing world, the problem of roaming dogs in urban areas is likely to increase. Frequent articles in the Indian national press, for example, reflect the current concern about rabies and dog bite incidents, in particular those involving children. Central to those discussions is the role of Animal Birth Control (ABC), which has been adopted in many Indian cities via the use of catch-neuter-release programmes directed at roaming dogs. Opinions are divided as to the effectiveness of such programmes in controlling the number of roaming dogs [e.g. [2,3]] yet the data needed to assess and optimise their effectiveness are largely lacking. Questionnaire surveys may provide demographic parameter estimates for owned dogs but little or no information on that component of the roaming dog population that is not owned. In this paper we suggest that, at little extra cost, data collection as part of the ABC programme itself can provide some of that missing information.

As part of an ABC programme run by Help in Suffering (HIS) in Jaipur since 1995, dogs are caught by trained HIS catching teams for spaying by complete ovariohysterectomy and rabies vaccination. To date a total of over 25,000 females have been spayed and vaccinated. Month to month variation in the proportion of spayed females that were pregnant has shown that the breeding cycle of dogs liable to catching by the catching teams is annual rather than biannual [4]. In this paper we derive a maximum likelihood (ML) estimate of the percentage of recruited females that become pregnant in any given year.

Spayed females are individually marked using a tattoo within the ear and a small notch is made in the ear margin to visibly mark the individual at a distance as having been spayed. Nevertheless spayed females are occasionally caught a second time when the ear notch is not noticed by the catching teams. Dogs are also caught for veterinary treatment or for euthanasia if they are terminally ill or injured, so if a tattoo marking is noted at that time the interval between first and second catching events is also available. We use the frequency distribution of those intervals to derive a ML estimate of the annual survival of spayed females.

Finally, we use estimates of litter size and age at first pregnancy from data collected in Jaipur [4] and a balance equation to calculate the probability of survival to age one for a female in a population that has reached carrying capacity and attained a stable age structure. Taken together these estimates are sufficient for a simple Leslie matrix model [5] of the Jaipur roaming dog population that can be used to assess the likely effect of future intervention.

## Results

### Fecundity

**Figure 1:**
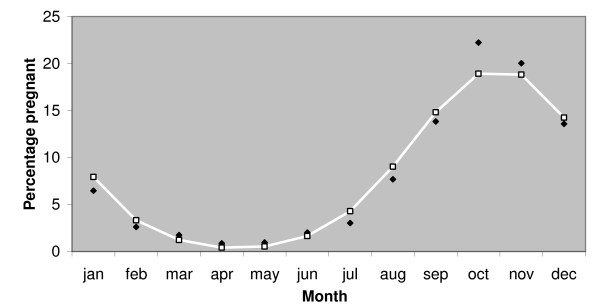
**Line graph showing observed pregnancy rates compared to expected**. Black diamonds show average percentage of females caught for sterilisation that were found to be pregnant by month. Females were not caught for sterilisation while lactating (within approximately 42 days following whelping) and pregnancy was detectable for approximately 56 days prior to whelping. White line shows expected percentage of detectably pregnant females caught monthly assuming 47.5% of recruited females become pregnant in any given year and whelping date is normally distributed about November 23^rd ^(± SD 58 days). Those parameter values maximise the likelihood of the observed percentages.

**Figure 2:**
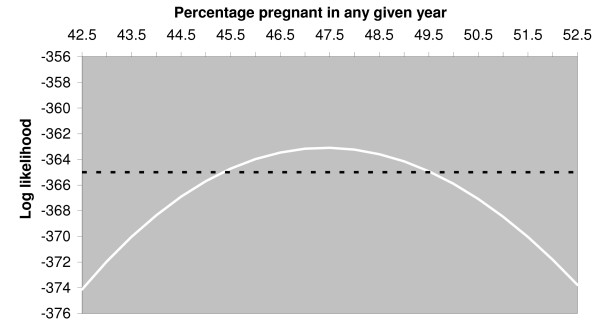
**Graph showing maximum likelihood curve for pregnancy rate estimate**. The continuous curve shows the change, with the percentage of females becoming pregnant in any given year, of the likelihood maximised with respect to the mean and standard deviation of whelping date. The dashed line is drawn at 1.92 below the maximum likelihood. Its intersection with the continuous curve provides 95% confidence limits for the estimate of the percentage of females becoming pregnant in any given year by exploiting the asymptotic chi-squared distribution of the likelihood ratio.

The ML estimate for pregnancy rate (i.e. the percentage of recruited females becoming pregnant in any given year) is 47.5%, with a 95% confidence interval from 44% to 51%. Whelping date was assumed to be normally distributed. The ML estimate for mean whelping date was November 23^rd ^with a standard deviation of 58 days. Figure 1 illustrates the observed month to month variation in the percentage of females collected for sterilisation that were found to be pregnant and the month to month variation in the expected value of that percentage given by the ML estimates. Figure 2 illustrates the variation, with pregnancy rate, of the likelihood maximised with respect to the mean and standard deviation of whelping date only and was used to derive the confidence interval on the pregnancy rate estimate.

### Survival

**Figure 3:**
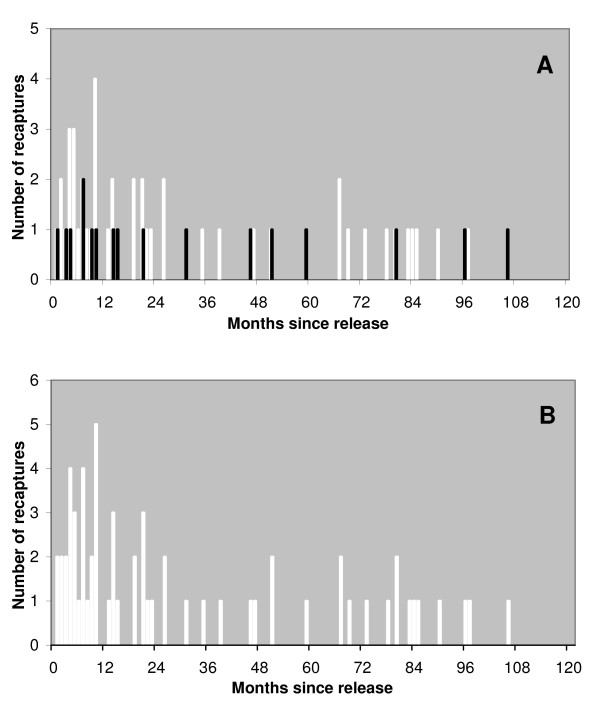
**a and b Frequency histograms for number of months between date released and recapture event**. The frequency distribution of time in months since the date of release for sterilised females that were caught a second time. In figure 3a white bars show the distribution for females caught a second time as a result of the ear notch having been missed, superimposed black bars show the distribution for females caught a second time because of terminal illness or injury. In figure 3b this data has been combined into one dataset as shown by the white bars.

**Figure 4:**
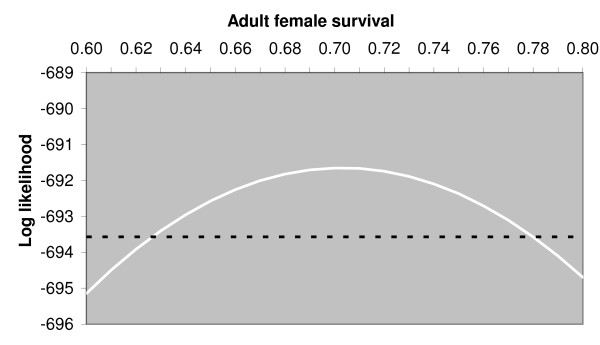
**Graph showing maximum likelihood curve for survival estimate**. The continuous curve shows the change, with adult female annual survival, of the likelihood maximised with respect to the probability per month of being caught a second time because the ear notch was missed and the probability per month of being caught a second time because of terminal illness of injury and a tattoo mark being recorded. The dashed line is drawn at 1.92 below the maximum likelihood. Its intersection with the continuous curve provides 95% confidence limits for the estimate of adult female annual survival by exploiting the asymptotic chi-squared distribution of the likelihood ratio.

The ML estimate for annual survival of spayed females that are at least one year old is 0.70 with a 95% confidence interval from 0.62 to 0.78. Figure 3a illustrates the frequency distribution of intervals prior to a spayed female being caught a second time because the ear notch was missed and compares that to the distribution of intervals prior to a spayed female being caught a second time because of terminal illness or injury. It suggests the two distributions are similar, so the survival rate estimate was based on the frequency distribution of intervals prior to all second catching events, illustrated in Figure 3b. In addition to the survival rate the likelihood function thus required parameters for the hazard rate (probability per month) of being caught as a result of the ear notch being missed and as a result of terminal illness or injury. Figure 4 illustrates the variation, with survival rate, of the likelihood maximised with respect to those two parameters only and was used to derive the confidence interval on the survival rate estimate.

### Juvenile survival

Assuming the population has reached carrying capacity and attained a stable age structure, the following balance equation provides an estimate of first year survival, *S*_*j*_, under those conditions:

$$S_{j}=\frac{1-\left(S/\lambda \right)}{f\cdot L\cdot \left({\left(S/\lambda \right)}^{r-1}-{\left(S/\lambda \right)}^{a}\right)}$$

where *f *is the pregnancy rate, *L *the average number of females per litter, *r *and *a *the ages at recruitment and senility (that is, the ages at which a female can have her first and last litters) and *λ *is the annual increase in the carrying capacity. We assume carrying capacity in Jaipur has increased in proportion to the human population at about 4% per year over the period of the ABC programme. From Chawla and Reece [4] the average litter size in Jaipur is 5.6 so, assuming an equal sex ratio at birth, *L *equals 2.8. Assuming that females are fully recruited to the breeding population at age 1 and do not become pregnant after age 7 the above expression gives a value of 0.25 for juvenile survival, *S*_*j*_, if the ML estimates of 0.70 and 0.475 are used for *S *and *f*. The calculated value of *S*_*j *_is insensitive to the assumed value of *a*, for example if females can become pregnant up to age 10 the estimate for *S*_*j *_is only slightly less at 0.24. Using estimates of 0.70 and 0.25 for *S *and *S*_*j *_gives a total expected lifespan of 1.3 years at birth and 3.8 years at one year old.

## Discussion

The World Health Organisation recognises that data on the ecology of street dogs are limited and that data collection needs to be extended to areas for which none exist [6]. The estimates reported here provide data for roaming dogs in a city that may be typical for north India in relation to dog demography.

Our values for the fecundity of roaming dogs in Jaipur are similar to published estimates. We estimate that in Jaipur 47.5% of female dogs aged one year and over have a litter in any given year. Pal [7] studied the ecology of roaming dogs on the outskirts of a town in West Bengal over a period of four years. He showed that breeding occurs over a single season per year and observed 77 litters. The average dogs density over the study period was 178 per km^2^, the study site covered 0.5 km^2 ^and the sex ratio was 1.37:1 males to females, so the number of litters as a percentage of the average number of adult females present was 51%. Butler and Bingham [8], working in rural Zimbabwe, state that a female had a litter every 1.6 years, equivalent to 0.6 litters per female per year. Kitala *et al *[9] present estimates from a questionnaire survey in Machakos District, Kenya. They report that 54% of female dogs had a litter during the year and an average fecundity of 1.3 female pups per female per year. Our estimate of pregnancy rate combined with the estimate of 2.8 females per litter from Chawla & Reece [4] gives an average fecundity of 1.33.

We have been unable to locate many comparable estimates for annual survival of roaming dogs in the urban environment. We estimate adult annual survival at 70% for spayed females. Pal [7] observed only 30 deaths over his four-year study period, suggesting an adult annual survival of over 91%. However the mean age at death observed for adult dogs (which he defined as being over 15 months old) was only 2.6 years. For dogs surviving to 15 months the mean age at death corresponding to our estimate of 70% adult annual survival is 4 years. This suggests that adult survival was lower than in Jaipur and that some deaths were not observed. His estimate of 18% for first year survival is also less than our estimate of 25% for first year survival when the Jaipur population was at carrying capacity, supporting his contention that his local population was being maintained by immigration.

Data from table 5 in Kitala *et al *[9] suggest a value of about 60% for adult female annual survival, again lower than our estimate, despite their observation that all dogs observed were owned. Beck [10] sets an upper bound of 77% on survival of roaming dogs in Baltimore by comparing the number of dogs known to have died over a year to the estimated total number in the city. Data in his table 4 give an estimate of 70% for annual survival of dogs at least one year old, thus identical to our estimate for spayed females at least one year old. However his data suggest that survival is not constant, increasing to a maximum of 87% for dogs from two to three years old and reducing thereafter. The same may apply to dogs in Jaipur but survival was assumed to be constant to allow for estimation by the method used.

Beran [11] noted that dogs less than one year old are over represented in canine rabies cases. Thus it should follow that controlling rabies in canine populations with limited reproductive performance, such as that of Jaipur, is easier than in populations with a more rapid turnover. Unsurprisingly the reproductive performance reported for pet dogs is much higher with lower pup mortality [12]. These authors note that litter size in bitches increases up to 3–4 years of age and is lower in young animals. The longevity figures reported here may be one reason why the fecundity of roaming females is low since these dogs are breeding at ages below the age at which they have largest litters.

Although the set of estimated parameters is necessarily simplified it is sufficient to establish an age-structured model for the female component of the Jaipur roaming dog population. The number of female spays is known accurately so the transfer of dogs from the fecund to sterile populations can be included in the model. The results thus provide the potential to generate a trajectory of population size, given a scenario of future spaying effort. Such a trajectory depends on how the intervention itself affects survival, age at first pregnancy and pregnancy rate. Although the data provide in principle the means to monitor changes in pregnancy and adult survival, the potential to estimate juvenile survival via the balance equation is no longer available once the population has been deflected to below carrying capacity. However, changes in the population structure observed since the outset of the ABC programme can be compared to the model output in order to place constraints on the parameter values. For example the percentage spayed can be observed as part of the intervention effort itself and hence monitored accurately without the need for extra resources.

HIS is currently using an age-structured model of the Jaipur roaming dog population based on these results to guide future intervention, however two main issues remain to be addressed in order to reduce uncertainty about the projections. One is how the roaming dogs that can be accessed by the catching teams differ in their survival and fecundity from those dogs the teams are unable to catch and the other is the degree to which spaying itself may increase female survival.

## Conclusion

The data used to derive the estimates presented here resulted from careful recording of information provided by the current intervention itself. Irrespective of information obtained prior to intervention, the fact that such data are able to generate usable estimates suggests that individual identification of the dogs treated, recording the time and location of each catching event and recording the reproductive condition of each dog is worthwhile and should form part of any monitoring programme used for evaluation. Furthermore, where the ABC programme involves catching dogs on the street, those involved in the catching process should be provided with the means to record the numbers of pups, males, females and lactating females encountered, with and without ear notches, as part of that process. Comparison of the observed structure of the population with that predicted by a simulation model will place constraints on the demographic parameter values. The number of ear-notched animals released is accurately known so, given an estimate of their rate of survival, their observed proportion in the population also allows the size of that population to be monitored as the intervention progresses.

## Methods

### The likelihood for the proportion of pregnant females by month

The females caught for sterilisation on a given day constitute a random sample of the females roaming the area designated for catching on that day. The number of pregnant females the sample is likely to contain depends on the proportion of recruited females that become pregnant in any given year, the timing of the breeding season in relation to the timing of catching and the spread of the breeding season over time. For example, the number is likely to be increased shortly before the peak of the breeding season. In this case, it will be further increased if the breeding season is of short duration and reduced if the season is more spread out. Thus the observed monthly variation in the proportion of females found to be pregnant (based on inspection of over 25,000 females caught for spaying from 1995 to 2006) provides information on fecundity. To estimate the proportion of females becoming pregnant in any given year we chose that proportion which, in conjunction with appropriate values for the timing and spread of the breeding season, mimics most closely the observed variation.

If *n*_*i *_females are collected in the *i*^th ^sample the number found to be pregnant, *k*_*i*_, has a binomial distribution

$$\begin{array}{c} n_{i} \end{array}C_{k_{i}}p_{i}^{k_{i}}{\left(1-p_{i}\right)}^{n_{i}-k_{i}}$$

where *p*_*i *_is the probability that a female collected in the *i*^th ^sample will be found to be pregnant. *p*_*i *_depends on *f*, the probability a recruited female becomes pregnant in any given year, which is the parameter of interest. It also depends on *μ *and *σ*, the mean and standard deviation whelping date, and on two further parameters: *D*_*p*_, the number of days prior to the whelping date for which a female can be seen, during the spaying operation, to be pregnant; and *D*_*l*_, the number of days following the whelping date for which a female can be seen to be lactating and will therefore not be collected for spaying. *D*_*p *_was set at 56 days and *D*_*l *_at 42 days. The probability a female collected in the *i*^th ^sample is found to be pregnant is the probability her next litter is due within *D*_*p *_days given that her last litter was not born less than *D*_*l *_days ago, thus

$$p_{i}=\frac{f\cdot \int_{t_{i}}^{t_{i}+D_{p}}N(t|\mu ,\sigma )dt}{1-f\cdot \int_{t_{i}-D_{l}}^{t_{i}}N(t|\mu ,\sigma )dt}$$

where *t*_*i *_is the date of the *i*^th ^sample expressed as days from the start of the year. To allow for the fact that, as a function of date, the variation in the proportion pregnant is cyclic, each normal density was replaced by the sum of the densities over the current, previous and following years and that sum scaled to integrate to one over a single year:

$$p_{i}=\frac{f\cdot \sum_{j=-1}^{j=1}\int_{t_{i}}^{t_{i}+D_{p}}N(t|\mu +j\cdot 365,\sigma )dt/\sum_{j=-1}^{j=1}\int_{1}^{365}N(t|\mu +j\cdot 365,\sigma )dt}{1-f\cdot \sum_{j=-1}^{j=1}\int_{t_{i}-D_{l}}^{t_{i}}N(t|\mu +j\cdot 365,\sigma )dt/\sum_{j=-1}^{j=1}\int_{1}^{365}N(t|\mu +j\cdot 365,\sigma )dt}$$

This expression for *p*_*i *_was used in the binomial distribution to calculate the probability of *k*_*i *_and those probabilities logged and summed over *i *to give the log likelihood of the observed variation in the proportion pregnant.

The asymptotic chi-squared distribution of a logged likelihood ratio was used to calculate confidence limits for the estimate of *f*. Figure 2 shows the change with *f *of the log likelihood maximised with respect to *μ *and *σ*. The horizontal line is drawn at 1.92 (i.e. *χ*^2^_1,0.05_/2) below the maximum of the log likelihood maximised with respect to all three parameters and the values of *f *at which it cuts the log likelihood curve provide lower and upper 95% confidence limits of 45.5% and 49.5% for the percentage of females that become pregnant in any given year. However, this calculation assumes that *D*_*p *_and *D*_*l *_are known without error whereas the period for which females can be seen to be lactating and are therefore not collected from the street is not known accurately. Allowing that period to vary from four to eight weeks reduces the lower confidence limit to 44% and increases the upper limit to 51%.

### The likelihood for the distribution of intervals preceding a second catching event

After being released back into the urban environment a small number of spayed females are caught by the catching teams a second time because the ear notch that identifies the female as having been spayed was missed. An even smaller number are recorded as having been caught a second time because they are terminally ill or injured and are therefore returned to the clinic for euthanasia.

The spayed females caught a second time provide a random sample of minimum times for which females persist in the population following being spayed and hence information on their survival rate. For example, if their survival was extremely low then almost all second catching events would occur soon after the operation. Data records were available from 2002 to 2006 inclusive. Over this period tattoo markings were recorded on about 0.6% of the adult female dogs caught, giving a sample size of 62 intervals between the first and second catching events. We used the proportion of females recorded as having been caught a second time and the distribution of time intervals to estimate the annual survival of recruited females.

Those statistics depend on the probability of survival in combination with the probabilities of the two types of catching in each successive month. Thus the likelihood of the observed proportion and intervals was maximised with respect to three parameters: the probability of survival, *S*; the probability per month of being caught a second time because of the ear notch having been missed, *R*_*h*_; and the probability of being caught and recorded a second time because of terminal illness or injury *R*_*s *_(only a proportion of dogs that were euthanised were checked for the presence of a tattoo mark).

The release of each sterilised female resulted in one of three types of event: released in a certain month and not caught a second time; first released in a certain month and caught a second time, after a certain interval, for euthanasia; and first released in a certain month and caught a second time after a certain interval because the ear notch was missed but not caught again. No females were recorded as caught more than twice.

The probability of the first type of event is the sum, over every month following the release, of the probability that the female survives until that month without being caught a second time and then dies in that month, plus the probability the female survives until the last month without being caught a second time. Let *n*_1*i *_represent the number of females released in month *i *that were not caught subsequently. For each of those females the probability that they were not caught subsequently was:

$$P_{1i}=\sum_{m=0}^{t-i-1}{\left(S(1-R_{h}-R_{s})\right)}^{m}(1-S)+{\left(S(1-R_{h}-R_{s})\right)}^{\left(t-i\right)}$$

where *t *is the final month in which dogs were caught.

The *n*_2*ij *_females released in month *i *that were caught for euthanasia in month *j *must have survived and avoided capture for *j-i *months before being caught, hence for each of them the probability of that event was:

*P*_2*ij *_= (*S*(1 - *R*_*h *_- *R*_*s*_))^(*j*-*i*) ^*R*_*s*_

The *n*_3*ij *_females released in month *i *that were caught because the ear notch was missed in month *j *must have survived and avoided capture for *j-i *months before being caught and then have avoided capture subsequently, hence for each of them the probability of that event was:

*P*_3*ij *_= (*S*(1 - *R*_*h *_- *R*_*s*_))^(*j*-*i*) ^*R*_*h *_*P*_1*j*_

Thus, assuming the probability of each event is independent, the log likelihood equals:

$$\sum_{i=1}^{f-1}n_{1i}\log(P_{1i})+\sum_{i=1}^{f-i}\sum_{j=i+1}^{f}n_{2ij}\log(P_{2ij})+\sum_{i=1}^{f-i}\sum_{j=i+1}^{f}n_{3ij}\log(P_{3ij})$$

*R*_*h *_and *R*_*s *_are taken as constants, so to avoid bias in the estimate of *S *it is necessary that, given a spayed female has survived to a certain month, the probability she is caught again in that month is independent of the period since spaying. That assumption could be violated if some spayed females had an increased mortality rate when first released and hence an increased probability of being recaptured early as dogs suffering from terminal illness or injury. In that case we would expect an excess of short intervals in the histogram of intervals relating to dogs caught for euthanasia. However figure 3a shows that the distributions of intervals prior to the two types of catching are very similar.

There is also a potential for changes in *R*_*h *_and *R*_*s*_over time to bias the survival estimate. The number of tattoo markings recorded as a percentage of the total number of females collected has been fairly constant since 2002 but there was some reduction in the effort to look for markings on dogs collected in 2004, when only half the usual number of marks were recorded. However, by that time the age distribution of spayed females in the population had stabilised and reducing the values of *R*_*h *_and *R*_*s *_to reflect the observed variation changed the survival estimate by less that 0.5%.

## Authors' contributions

JR and SC manage the intervention on which the research is based, ensure accurate collection of data associated with the intervention, collated the data for this analysis and provided interpretation of analysis results. EH identified analysis needs, provided interpretation of analysis results and critical review of manuscript. LH designed and carried out analysis of data, interpreted analysis results and drafted the manuscript. All authors read and approved the final manuscript.

## References

[B1] Nassar R, Mosier JE (1980). Canine Population Dynamics: A Study of the Manhattan Kansas, Canine Population. American Journal of Veterinary Research.

[B2] Reece JF, Chawla SK (2006). Control of rabies in Jaipur, India, by the sterilisation and vaccination of neighbourhood dogs. The Veterinary Record.

[B3] Sudarshan MK, Nagaraj S, Savitha B, Veena SG (1995). An epidemiological study of rabies in Bangalore city. Journal of Indian Medical Association.

[B4] Chawla SK, Reece JF (2002). Timing of oestrus and reproductive behaviour in Indian street dogs. Veterinary Record.

[B5] Leslie PH (1945). On the Use of Matrices in Certain Population Mathematics. Biometrika.

[B6] Anon (2004). WHO expert consultation on rabies: first technical report. Technical Report Series 931.

[B7] Pal SK (2001). Population ecology of free-ranging urban dogs in West Bengal, India. Acta Theriologica.

[B8] Butler JRA, Bingham J (2000). Demography and dog-human relationships of the dog population in Zimbabwean communal lands. Veterinary Record.

[B9] Kitala P, McDermott J, Kyule M, Gathuma J, Perry B, Wandeler A (2001). Dog ecology and demography information to support the planning of rabies control in Machakos District, Kenya. Acta Trop.

[B10] Beck AM (1973). The ecology of stray dogs: A study of free-ranging urban animals.

[B11] Beran GW, Baer GM (1991). Urban rabies. The Natural History of Rabies.

[B12] Linde-Forsberg C, Eneroth A, Simpson GM, England GCW, Harvey M (1998). Parturition. The Manual of Small Animal Reproduction and Neonatology.

